# Supplementary material for: MetaRibo-Seq measures translation in microbiomes
Source: Nat Commun. 2020 Jun 29;11:3268. doi: 10.1038/s41467-020-17081-z (PMC7324362; doi:10.1038/s41467-020-17081-z)
Supplement: Supplementary file 10 — Supplementary Data 7 [file 41467_2020_17081_MOESM10_ESM.zip › File2/Confidence_High_Taxonomy/148462_out.krona.html]

Javascript must be enabled to view this page.

members
magnitude
magnitudeUnassigned
count
unassigned
taxon
rank

148462\_out

3

superkingdom
3
2

1239
3
phylum

186801
class
3

186802
3
order

3
family
186803

189330
genus
3

3
species

SRS105153\_contig\_number\_contig-100\_29426.29426SRS142712\_contig\_number\_contig-100\_22628.22628SRS146812\_contig\_number\_30677
88431
